# Unseen Faces, Lingering Storylines

**DOI:** 10.3201/eid2313.AC2313

**Published:** 2017-12

**Authors:** Byron Breedlove

**Affiliations:** Centers for Disease Control and Prevention, Atlanta, Georgia, USA

**Keywords:** art science connection, emerging infectious diseases, art and medicine, about the cover, Alan Mermin-Bunnell, 28,616, unseen faces, lingering storylines, Ebola, Ebola virus, epidemic, pandemic, global health security, West Africa

**Figure:**
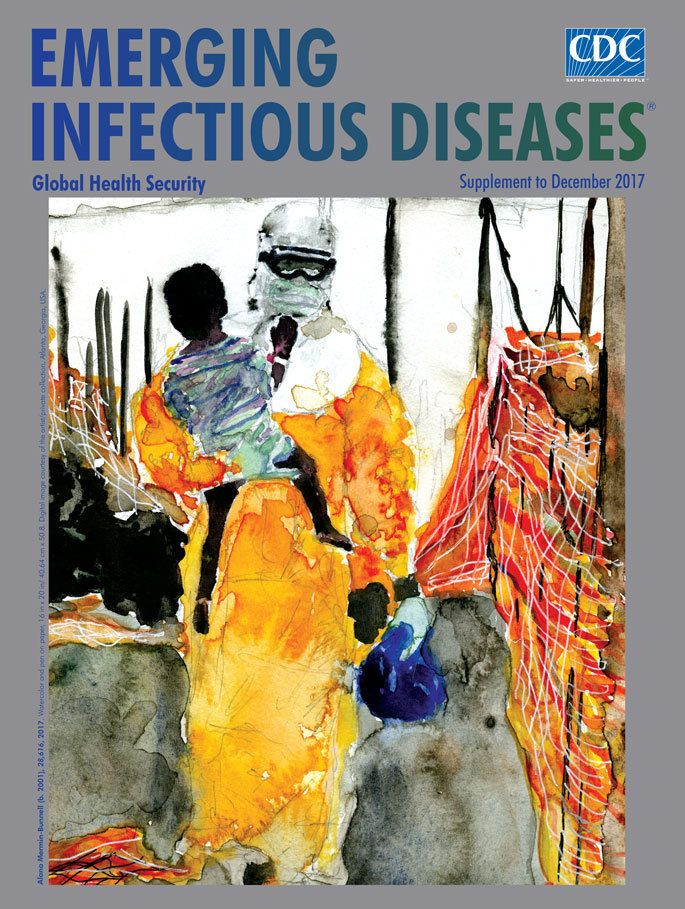
**Alana Mermin-Bunnell (b. 2001), 28,616, 2017.** Watercolor and pen on paper, 16 in × 20 in/40.64 cm × 50.8 cm. Digital image courtesy of the artist/private collection, Atlanta, Georgia, USA.

Unlike previous outbreaks of Ebola virus disease that occurred in difficult to reach rural settings, the 2014–2016 West Africa Ebola epidemic involved major urban areas and thereby increased the potential to escalate from a regional epidemic to a pandemic. The West Africa Ebola outbreak was unprecedented in its scope, resulting in 28,616 reported cases and 11,300 deaths among adults and children. In all, Ebola cases were treated in 10 different countries during this outbreak. 

Approximately 800 confirmed and suspected cases of Ebola virus disease occurred among healthcare workers. According to the World Health Organization, these workers had a “critical yet high risk role in responding to the Ebola epidemic and in working to meet the health needs of their communities during the epidemic. Many paid for this with their lives.”

The title of this supplement issue’s cover art, *28,616*, by American artist Alana Mermin-Bunnell, corresponds to the number of Ebola cases that were reported during the 2014–2016 West Africa epidemic. The painting depicts one moment during the outbreak. The artist uses a pallet of bright watercolors dominated by searing yellows, reds, and oranges that evoke both the urgency of the response and the caution essential to protect responders and patients. 

A healthcare worker sheathed in yellow personal protective equipment (hood, goggles, and gloves) commands the viewer’s attention. Standing before one of the Ebola treatment centers built in West Africa during the international public health response, this unknown worker clutches a young child to his or her shoulder with one hand and grips a bright blue bag in the other. The healthcare worker looks toward the ground, shoulders slumped, while walking between strands of plastic barrier fencing that look like a bright tapestry sagging under relentless sun and heat. 

Multiple questions and potential storylines converge in this painting. Some are simple curiosity. What is inside the blue bag the healthcare worker clutches? Why is the child wearing a medical bracelet on his or her right wrist? Is the child being quarantined and separated from an infected parent sequestered in the treatment center? Did the child and his or her parents survive? The image also raises other complex questions. For instance, how is the responder handling the ethical and moral dilemmas that arise from decision making with regard to triage, quarantine, surveillance, and burial rituals and ceremonies? How is the local population reacting to restrictions and disruptions? Are these reactions endangering the public health responders and community and villages? How are responders handling the task of following up with people who may have been in contact with an individual with Ebola?

Mermin-Bunnell notes, “The Ebola epidemic forced a layer of separation between caretaker and patient. My painting, of a person in PPE (personal protective equipment) holding a child, tries to convey the humanity and emotion of healthcare workers that has been evident throughout the epidemic despite the dehumanizing use of PPE.” (All quotes from A. Mermin-Bunnell, pers. comm., 2017 Aug 9) The faces of the child and the healthcare worker are unseen, making the viewers guess at what their emotions are at this moment. 

The artist had a direct, personal connection to the events in West Africa. During the fall of 2014, both of her parents were deployed to Sierra Leone to fight the Ebola epidemic. That experience, she explained, “was transformative for our family, and I gained a greater perspective of the process of fighting for global health security. There are sides to the effects of the Ebola epidemic that are harder to see than the obvious tragic death toll.” 

The West Africa Ebola epidemic brought heightened awareness of the need to enhance health security in all countries worldwide. The late D.A. Henderson wrote, “Today, cases and outbreaks of disease, whatever their cause and wherever they occur, pose a threat to people throughout the world. No major city in the world is more than 36 hours distant from any other.” A lingering storyline from this issue’s cover art from the West Africa Ebola outbreak is the need to strengthen global health security should the next epidemic become a pandemic and potentially overwhelm infrastructures, cripple economies, and claim millions of lives. 

Pandemics have been a dark part of global history. The Black Death, an outbreak of bubonic plague that occurred during 1346–1353, was the most devastating pandemic in history and “swept away around 60 per cent of Europe’s population” according to researcher Ole Jørgen Benedictow. The Spanish flu emerged during the final phases of World War I, during 1918–1920, and spread throughout the world, killing between 50 and 100 million people, and sickening more than 500 million people. 

To protect the world from infectious disease threats, public health agencies, nations, international organizations, and public and private stakeholders need to cooperate and coordinate to prevent and reduce outbreaks, detect threats, and respond effectively. Those unseen faces and lingering storylines from the painting *28,616* remind us of what is at stake. 
